# Reviewed article: Araújo LBS, Sousa RA, Aguiar BGA, Mendonça VJ, Araújo OD, Tajra FS. Spatial and temporal distribution of neglected tropical diseases: overlap analysis, Piauí, 2013-2022. Epidemiol Serv Saude. 2026:35;e20250373

**DOI:** 10.1590/S2237-96222026v35e20250373.d

**Published:** 2026-03-16

**Authors:** Marilia Mastrocolla de Almeida Cardoso

**Affiliations:** 1 Ministério da Saúde, Brasília, DF, Brazil

## First round comments

Prezados autores, em complemento ao que foi apontado pelos revisores, seguem abaixo algumas sugestões:

Resumo

Deve haver um alinhamento entre o título, objetivo e método. No título está “Distribuição espacial e temporal”, enquanto que no objetivo está “Analisar a tendência temporal, caracterizar a magnitude, o perfil sociodemográfico e a distribuição espacial das doenças tropicais negligenciadas com sobreposição no Piauí, entre 2013 e 2022” e no método “Análise série temporal, quantitativa e analítica”

Introdução

Inserir dados que reforçam a importância de se realizar uma análise da distribuição espacial e temporal no contexto do estudo e o impacto/contribuição para o SUS

Método

Remover financiamento do texto. As informações devem ser inseridas somente na plataforma. 

Qual tipo de média foi utilizada? Aritmética ou ponderada, considerando a diferença de números de

habitantes em cada município.

Resultados

Falta o “que” nessa sentença: Além disso, foi verificado ( ) a doença de Chagas, diferentemente das outras doenças tropicais negligenciadas, acometeu mais as pessoas do sexo feminino (51,1%) e residentes da região de saúde do Vale Rio Guaribas (32,2%) (Tabela 1).

Rever os títulos da tabelas

Padronizar “n=”.

Discussão

Como já mencionado por um dos revisores, será necessário inserir as limitações do estudo.

Recommendation: Major revision

## Second round comments

Prezados autores, o documento necessita de pequenos ajustes:

1- Tabela 1: ajustar a variável faixa etária para: “Faixa etária (anos)”.

2- O texto da “Disponibilidade dos dados” ainda precisa de ajustes. Não há necessidade de citar a fonte de onde os dados foram coletados, como está colocado no documento (“Os dados utilizados foram solicitados e disponibilizados pela Secretaria do Estado de Saúde do Piauí, através do site ttps://site.saude.pi.gov.br/ e os códigos utilizados estão disponibilizados pela 10ª revisão da Classificação Internacional de Problemas Relacionadas às Doenças (CID-10).”)

No texto, os autores devem mencionar a forma de acesso aos dados de pesquisa (bancos de dados gerados para análise, códigos, métodos e outros materiais utilizados e resultantes da pesquisa), que poderá ser feito via link do repositório e referenciamento, com a devida citação no texto ou mediante solicitação aos autores.

Recommendation: Minor revision

